# Evaluation of component alignment in total knee arthroplasty using patient-specific instrumentation versus conventional guides: a retrospective study

**DOI:** 10.1051/sicotj/2025044

**Published:** 2025-08-04

**Authors:** Georgios Renieris, Athanasios Georgokostas, Eleni Georgaki, Natalia Renieri

**Affiliations:** 1 Orthopedic Department, Athens Medical Group, Psychiko Clinic Andersen 1 Athens 11525 Greece; 2 Athens Orthopedic and Trauma Clinic Athens 11525 Greece; 3 Oncology Unit, General Hospital of Athens Hippocrateion Athens 11527 Greece

**Keywords:** Total knee arthroplasty, Alignment, Implants, Patient-specific instrumentation, Customized, Conventional

## Abstract

*Background*: To evaluate whether the use of patient-specific instrumentation (PSI) or conventional instrumentation (CI) is associated with superior implant positioning and knee alignment in total knee arthroplasty (TKA). *Methods*: Clinical data, pre- and post-operative knee X-rays of 95 patients, who underwent TKA with use of either patient-specific instrumentation (group PSI) or conventional intra-/extramedullary cutting guides (group CI) were retrospectively collected. Preoperative measurements of knee alignment were done by assessing the femorotibial axis, the lateral femoral distal angle, and the medial tibial proximal angle. Postoperative measurements of the mechanical TKA alignment were performed by assessing the relative position of components to the femur and tibia and the femorotibial axis angle. Only when all three parameters were within generally accepted limits was the postoperative radiological outcome considered optimal. *Results*: Preoperative measurements and demographics were similar among the two groups. No statistically significant differences were found between postoperative radiographic findings in patients operated on with PSI or CI. A restoration of the femorotibial axis was achieved in 87.8% and 87.0% of patients treated with PSI and CI, respectively (*p* = 0.583). Coronal alignment of the femoral component was within acceptable limits in 97.6% and 94.4% (*p* = 0.631) of patients of the PSI and CI groups, respectively. The respective percentages for the tibial component were 85.3% and 83.3% (*p* = 0.510) of patients. An accurate coronal plane radiological outcome was achieved in 82.9% and 77.8% of patients treated with PSI and CI, respectively (*p* = 0.611) *Conclusions*: The use of PSI does not increase the accuracy of component positioning and leg axis restoration compared to CI in TKA in patients with mild deformity.

## Introduction

Primary total knee arthroplasty (TKA) has become one of the most frequently performed orthopaedic procedures, with 1,899,847 cases reported between 2012 and 2023 in the American Joint Replacement Registry [1]. According to the UK National Joint Registry, all-cause survivorship of TKA at 10 and 15 years is 96.7% and 95.4%, respectively [2].

Despite increasing numbers of knee arthroplasties and advancements in implant design, around 20% TKA patients remain dissatisfied [3]. Implant malalignment has been linked to poorer patient-reported outcomes and has been reported to lead to a 2-fold to 3-fold increase in the failure rates of TKAs [4]. Alignment strategies include the a) anatomical, b) mechanical, and c) kinematic alignment. Mechanical alignment has been shown to equalize the load on the medial and lateral compartments of the femoral and tibial components of the prosthesis, thus leading to decreased component wear and risk of loosening and increased TKA survival rates [4].

Traditionally, the bone cuts performed for the restoration of the mechanical alignment of the joint have been performed with the use of conventional instrumentation (CI), which is aligned through intra-extramedullary guiding rods to the anatomic axis of the femur and tibia. However, several authors have proposed, to address the issue of malalignment, the use of personalized realignment strategies, through patient-specific instrumentation (PSI). Patient-specific implants are designed based on pre-operative computerized tomography (CT) or magnetic resonance imaging (MRI) to produce a 3-D image and subsequently construct individualised 3-D printed cutting guides.

Several studies have compared the functional outcomes after TKA using PSI or CI with controversial results [5]. However, evidence on the actual differences in TKA alignment when PSI or CI are used is lacking. This study aimed to evaluate whether the use of PSI leads to a superior implant positioning and knee alignment compared to CI in TKA.

## Patients and methods

### Patient selection and data acquisition

Our database was used to retrieve data of all patients who underwent TKA due to end-stage knee osteoarthritis between January 2022 and December 2024 in our orthopedic department.

A non-randomized retrospective study was designed. Inclusion criteria were a) patients, male and female, who underwent mechanically aligned (MA) primary TKA using a cruciate retaining (CR) prosthesis, with use of either PSI or CI, for end-stage knee osteoarthritis. Exclusion criteria were a) posttraumatic osteoarthritis; b) severe arthritic bone loss; c) flexion contracture > 10° and d) axis deformities over 20°. All enrolled patients provided their informed consent.

### Surgical technique

All TKA procedures were performed by the same surgical team, consisting of three experienced orthopedic surgeons.

TKAs were performed using either PSI (a) Persona/Signature (Zimmer, Warsaw, IN, USA); b) ACS One Fit (Implantcast, Buxtehude, Germany) or CI (a) Persona/Signature (Zimmer, Warsaw, IN, USA); b) Sigma Total Knee System (DePuy Synthes Johnson & Johnson, East West Chester, PA, USA). PSI was designed based on a preoperative full leg computer tomography performed 1 month prior to surgery. Preoperative planning in the conventional TKA group was performed on a full leg AP X-ray according to the Knee Society recommendations [6].

A midline incision and a medial parapatellar approach were used. For patients in the PSI group, resection of the proximal tibia and distal femoral were performed using the customized cutting guides. For patients in the CI group, tibial resection was performed using an extramedullary guide with a posterior slope according to the manufacturer’s instructions. Distal femoral resection was conducted using an intramedullary guide, with a valgus angle 4–6°. For valgus knees, a reduced angle of 3° was preferred. Soft tissue release was performed, if necessary, to achieve a symmetrical extension gap. After femoral sizing, femoral preparation with the relevant 4-in-1 cutting block was conducted. Assessment of extension and flexion gap. Sizing, establishment of rotation of the tibia, and then the tibia was drilled and broached. Stability was verified prior to implanting the components, which were fixed using tobramycin-laden cement.

### Radiographic evaluation

For the purposes of this study, in addition to the standardized imaging utilized for the preoperative planning, a weight-bearing AP radiograph and lateral radiograph of the knee were obtained preoperatively. Immediately postoperative AP and lateral knee radiographs were also obtained. Radiographs were performed following recommended protocols [7].

**Figure 1:**
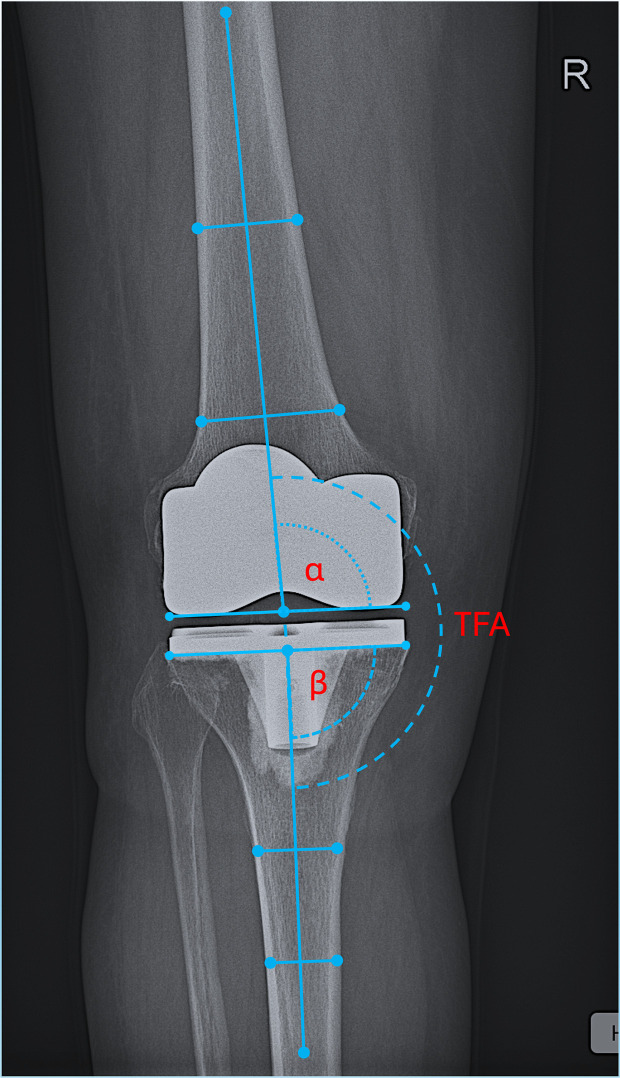
Radiographic measurements of TKA component placement in the coronal plane. Limb coronal alignment is measured by the medial tibio-femoral axis angle (mTFA), defined as the medial angle between the anatomic femoral and tibial axes. A restored neutral mechanical alignment in the coronal plane was considered when the mTFA was 185° ± 3°. Femoral component placement in the coronal plane is measured by the α angle, defined as the angle formed between a line formed by the femoral condyles of the femoral component and the anatomical femoral axis. An α angle of 95° ± 3° represents a correct placement of the femoral component in the coronal plane. Tibial component placement in the coronal plane is measured by the β angle, defined as the angle formed between the tangent line at the base of the tibial component and the anatomical tibial axis. A β angle of 90° ± 3° represents a correct placement of the tibial component in the coronal plane.

**Figure 2:**
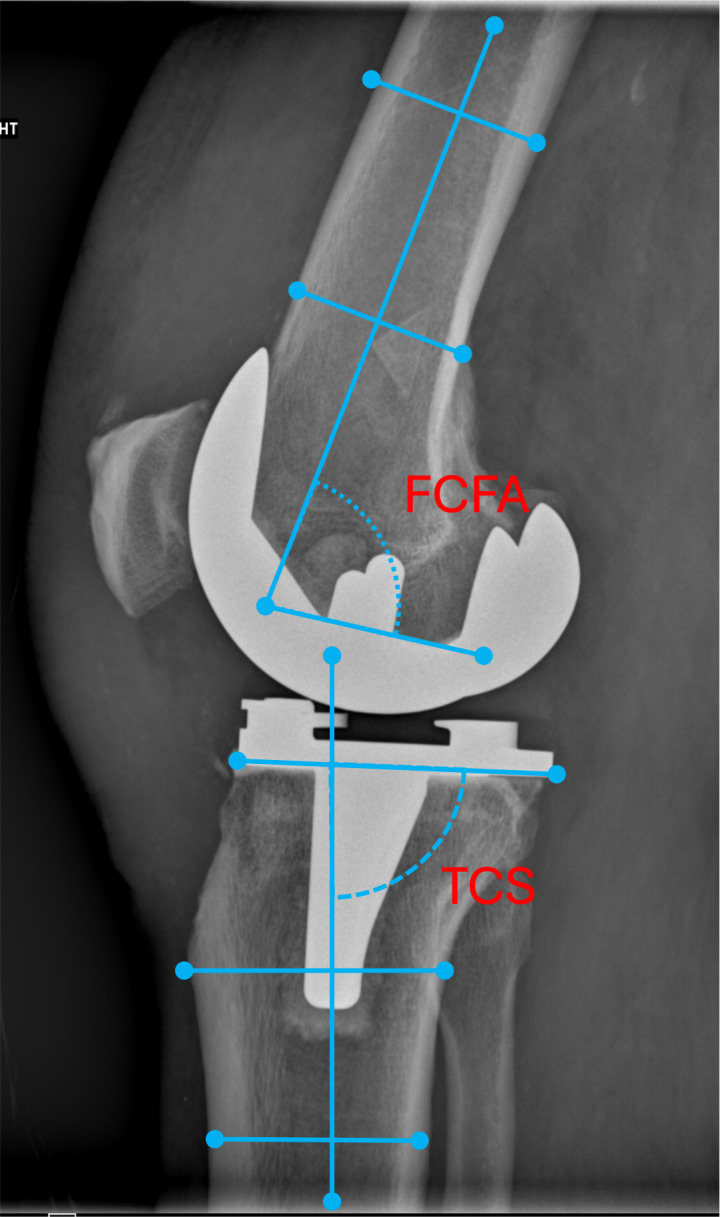
Radiographic measurements of TKA component placement in the sagittal plane. In the sagittal plane, the femoral component is assessed by femoral component flexion angle (FCF), defined as the angle between the distal femoral axis in the sagittal plane and a line perpendicular to the distal surface of the femoral component. A femoral component flexion angle of 0–8° is usually aimed. Tibial component placement is assessed by tibial component slope (TCS), defined as the angle between the line that is perpendicular to the bottom of the tibial plate and the tibial shaft axis in the sagittal plane. TCS measurements must consider that some TKA types have the posterior slope built into the implant design.

Alignment evaluation preoperatively included measurement of the lateral tibio-femoral angle (TFA), defined as the angle formed between the tibial and femoral anatomic axis. The anatomic femoral axis (aFA) was defined by the Moreland method, and the anatomic tibial axis (aTA) by the Oswald method. The anatomic lateral distal femoral angle (aLDFA), defined as the lateral angle formed between a line at the femoral condyles and the anatomical femoral axis, as well as the medial proximal tibial angle (mPTA), defined as the medial angle between the tangent at the tibial plateau line and the anatomical tibial axis, were measured. Last, the femoral condyle tibial plateau angle (FCTP), defined as the angle between a tangential to the femoral condyles line and a line tangential to the tibial plateau, was evaluated as an indicator for the pattern of osteoarthritis [8]. On postoperative AP radiographs a) the tibio-femoral axis angle (TFA); b) the alpha angle, defined as the angle formed between a line formed by the femoral condyles of the femoral component and the anatomical femoral axis and c) the beta angle, defined as the angle formed between the tangent line at the base of the tibial component and the anatomical tibial axis were measured (Figure 1). In the sagittal plane, the femoral component flexion angle (FCF), defined as the angle between the distal femoral axis in the sagittal plane and a line perpendicular to the distal surface of the femoral component, was already measured (Figure 2) [7]. A radiographic evaluation of the tibial component slope (TCS), defined as the angle between the line that is perpendicular to the bottom of the tibial plate and the tibial shaft axis in the sagittal plane, was not performed, as some of the TKA types used have the posterior slope built into the implant design.

As target values for a restored neutral mechanical alignment in the coronal plane, we considered an mTFA of 185° ± 3°, an alpha angle of 95° ± 3,° and a beta angle of 90° ± 3°. An accurate radiological outcome of TKA was considered only when all three parameters were within limits. In the sagittal plane, a femoral component flexion angle of 0–8° was deemed as correct [9].

All radiographic evaluations were performed independently by the three orthopaedic surgeons, who were blinded to patient allocation. The mean value of the three measurements was used in the statistical analysis.

### Statistical analysis

Categorical data were presented as frequencies, and quantitative characteristics were expressed as mean values ± standard deviation (SD). Univariate analysis between groups was performed with Fisher’s exact test for qualitative variables and the student’s *t*-test for independent values. Any *p*-value below 0.05 was considered statistically significant.

## Results

Between January 2022 and December 2024, 95 patients who met the inclusion criteria underwent primary TKA. TKA using PSI was performed on 41 patients (43%), whereas CI was used for the rest of the patients. Patients of both groups showed similar demographic characteristics (Table 1).

**Table 1:** Baseline characteristics and preoperative radiographic alignment parameters of patients who underwent TKA using patient-specific or conventional instrumentation.

	PSI (*n* = 41)	CI (*n* = 54)	*p*-value
Age (years, mean ± SD)	72.9 ± 6.3	73.0 ± 9.1	0.018**
Male gender (*n*, %)	13 (31.7)	17 (31.5)	0.577*
BMI > 30 (*n*, %)	9 (16.7)	6 (14.6)	0.510*
Valgus OA (*n*, %)	4 (9.8)	10 (18.5)	0.157*
Alignment parameters (degrees, mean ± SD)			
TFA	178.31 ± 4.66	177.58 ± 7.78	0.635**
aLDFA	82.03 ± 2.13	82.24 ± 2.42	0.697**
mPTA	86.11 ± 3.44	86.64 ± 4.31	0.576**
FCTPA	10.17 ± 7.64	8.70 ± 8.99	0.466**
TS	6.06 ± 1.95	5.27 ± 2.08	0.112**

Abbreviations: PSI: patient-specific instrumentarium; CI: conventional instrumentarium; BMI: body mass index; OA: Osteoarthritis; TFA: tibio-femoral angle; aLDFA: anatomic lateral distal femoral angle; mPTA: medial proximal tibial angle; FCTPA: femoral condylar-tibial plateau angle; TS: tibial slope; SD: standard deviation. *Comparisons by Fisher’s exact test; **Student’s *t*-test.

Preoperatively, the most common pattern observed in both groups was varus osteoarthritis, indicated by an FCTPA > 0° (90.2%; *n* = 37 for the PSI group and 81.5%; *n* = 44 for the CI group, *p* = 0.762). aLDFA was within normal range in 31 (75%) and 38 (70%) of patients of the PSI and CI groups, respectively (*p* = 0.788). mPTA was within normal range in 28 (68%) and 33 (90%) of patients of the PSI and CI groups, respectively (*p* = 0.461). There were no statistically significant differences in the preoperative radiographic measurements between patients treated with PSI and CI (Table 1).

**Figure 3:**
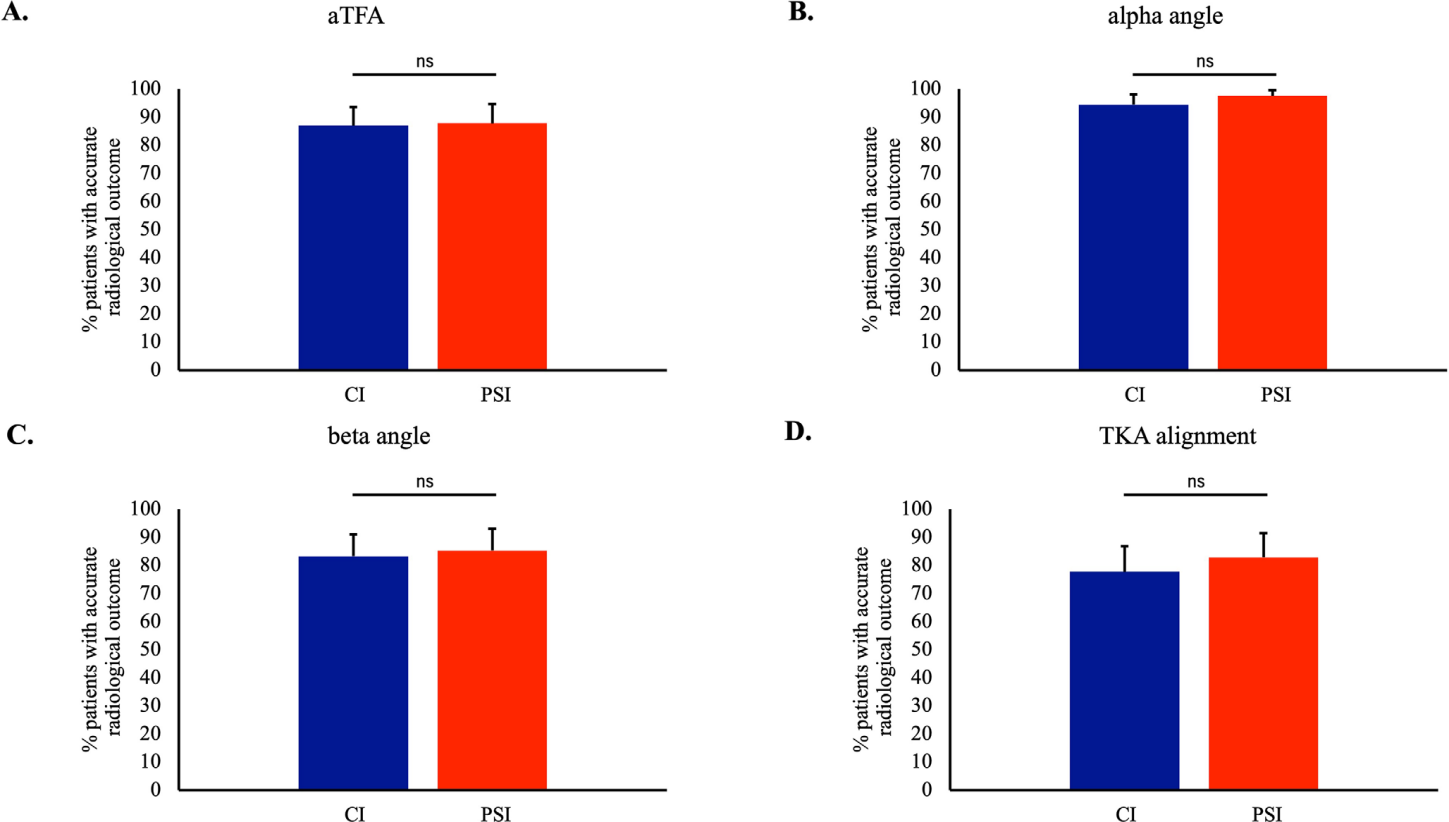
Comparison of radiographic parameters of limb alignment and TKA component placement in patients treated with PSI and CI. Radiographic parameters of limb alignment and component placement were measured on anteroposterior knee-rays of patients who underwent TKA with the use of conventional instrumentation (CI) or patient-specific instrumentation (PSI). A) The medial tibio-femoral axis angle (TFA) was measured in anteroposterior knee X-ray and was considered accurate when it lay between 182° and 188°. B) Femoral component placement in the coronal plane was assessed by the α angle and was considered accurate when the α angle lay between 92° and 98°. C) Tibial component placement in the coronal plane was assessed by the β angle and was considered accurate when the β angle lay between 87° and 93°. D) Accurate radiological outcome was considered when the TFA, the α angle, and the β angle were measured within respective limits. Comparison by Fisher’s exact test; ns: non-significant.

Postoperative mTFA was 183.29° ± 1.93° for the PSI group and 183.2° ± 2.02° for the CI group. Mean alpha and beta angles were 95.27° ± 1.87° and 88.07° ± 1.81° for the PSI group. The respective angles for the CI group were 95.24° ± 1.91° and 88.04° ± 1.94° (Table 2). A restoration of the coronal mechanical alignment, as indicated by a mTFA of 186° ± 3°, was achieved in 87.8% (*n* = 36) and 87.0% (*n* = 47) of cases treated with PSI and CI respectively (*p* = 0.583). A correctly implanted femoral component (alpha angle 96° ± 4°) was achieved in 97.6% (*n* = 40) and 94.4% (*n* = 51) of cases treated with PSI and CI, respectively (*p* = 0.631). The respective percentages for a correctly implanted tibial component (beta angle 90° ± 3°) were 85.3% (*n* = 35) and 83.3% (*n* = 45) (*p* = 0.510). An accurate coronal plane radiological outcome, indicated by all three parameters lying within the expected value range, was achieved in 82.9% (*n* = 34) and 77.8% (*n* = 42) of patients treated with PSI and CI, respectively (*p* = 0.611) (Figure 3).

**Table 2:** Postoperative radiographic alignment parameters of patients who underwent TKA using patient-specific or conventional instrumentation.

	PSI (*n* = 41)	CI (*n* = 54)	*p*-value
Alignment parameters (mean ± SD)			
TFA	183.29 ± 1.93	183.22 ± 2.02	0.864**
Alpha angle	95.27 ± 1.87	95.24 ± 1.91	0.944**
Beta angle	88.07 ± 1.81	88.04 ± 1.94	0.926**
FCF	0.46 ± 2.67	0.87 ± 1.29	0.330**

Abbreviations: PSI: patient-specific instrumentarium; CI: conventional instrumentarium; TFA: tibio-femoral angle; FCF: femoral component flexion; SD: standard deviation. Alpha angle represents the anatomic medial distal femoral angle; Beta angle represents the anatomic proximal medial tibial angle. **Comparisons by student’s *t*-test.

Additionally, in the sagittal plane, the femoral component flexion angle was similar between groups (0.10 ± 2.90 in the PSI group and 0.81 ± 1.36 in the CI group, *p* = 0.112). A correct femoral component sagittal positioning was found in 87.8% (*n* = 36) and 98.1% (*n* = 53) of patients treated with PSI and CI, respectively (*p* = 0.081). These results suggest that PSI does not increase the radiographic accuracy of placement of TKA components compared to CI.

## Discussion

This study aimed to evaluate the possible advantages of the use of PSI over CI in achieving optimal limb alignment and especially component positioning.

Although primary TKA is very effective in pain reduction and increasing knee function, patient dissatisfaction rates remain high. Implant malalignment of primary TKA is considered a significant risk factor for revision [10] and inferior patient-reported outcomes [11]. Data support that the correct alignment in the coronal plane and not in the sagittal plain is crucial for functional outcomes and implant survival [12].

The best strategy for alignment optimization in TKA has long been debated. Three strategies (anatomic, mechanical, and kinematic) are generally accepted to obtain an “optimal” component alignment in TKA [13]. The mechanical alignment, originally described by John Insall, aims at positioning the femoral and tibial components perpendicular to the femoral and tibial mechanical axis, respectively, in order to evenly distribute the joint loading forces [14]. Data concerning the superiority of the forementioned alignment strategies have been controversial. Nevertheless, mechanical alignment is still considered the gold standard in primary TKA [15] and it was preferred in our study.

Moreover, the optimal TKA alignment itself has become a subject of confrontation. In general, as a neutral overall coronal alignment, a 2–8° valgus aTFA should be targeted. Concerning femoral component placement, the aim is a 2–8° coronal valgus with respect to the FAA, and the tibial component should be placed in neutral coronal alignment at 90°. In the sagittal plane, the femoral component should be placed with 0–3° of flexion, and the tibial slope should be 0–7°, always in respect with the manufacturer’s instructions [9].

The use of PSI has been suggested to optimize limb alignment in TKA, improve surgical outcomes, and patient satisfaction [16]. Concerning patient-reported results, the results have been contradictory. Some small cohort studies have suggested an improved functional outcome of PSI-guided TKA compared to TKA using conventional instruments [17]. These studies have shown a slight but significant difference in the improvement of functional scores. On the other hand, several studies have failed to verify these differences, concluding that the routine use of PSI should not be recommended due to the higher cost and the waiting time [18]. In a 2019 meta-analysis, data from a total of 3,478 patients from 38 studies (including RCTs and non-RCTs) were analyzed [19]. The authors concluded that TKA-PSI does not improve patient-reported outcome measures, surgery time and complication rates as compared to standard TKA. A meta-analysis in 2022 [6], which included 23 randomized controlled trials with 2277 patients undergoing TKA with PSI or CI, showed no statistically significant differences in functional outcomes at 3-, 6-, and 12-month follow-up.

Data on the effect of PSI on the radiographic results and limb alignment after TKA are lacking. Our study showed that the use of PSI did not significantly improve coronal limb alignment compared to CI, as no statistically significant differences in mean aTFA could be found. Moreover, no statistically significant differences in femoral component positioning in the coronal (alpha angle) and sagittal (femoral component flexion) plane could be found among patients of the two groups. Last, coronal alignment of the tibial component (beta angle) did not statistically differ between groups. The number of outliers from optimal positioning was not statistically different between PSI-TKAs and CI-TKAs. Our results are in accordance with the limited existing data, which suggest that radiological accuracy was not significantly improved using PSI [20]. However, some contradict that the negative results of these studies could be associated with the use of old data and older technologies and may not reflect the effectiveness of the currently available technology [21]. Therefore, we believe that studies including the use of new-generation PSI, such as this one, are important for further evaluating the potential of new technologies in joint reconstruction.

Although the results of this study are not in favour of PSI over CI, they should not be interpreted as a suggestion against using PSI. The impact of several surgeon-specific factors on the effectiveness of conventional or PSI technology must be taken into consideration. The training status and the volume of operated patients per year are considered most important. Experienced surgeons with significant numbers of performed TKAs per year (>50 per year) have been reported to achieve similar radiological results after TKA, using PSI or CI [22]. PSI has been shown to help surgeons in training to achieve similar radiological accuracy to experienced surgeons [23].

It must be stressed that PSI has gained attention particularly for patients presenting with substantial coronal or sagittal plane deformities or extra-articular deformities [24, 25]. The mild nature of deformities in the study population may have diminished the alignment advantage associated with PSI. One additional indication for the use of PSI would be in patients with a pre-existing implant occupying the medullary canal, thereby precluding the use of standard intramedullary instrumentation.

## Conclusion

Patient-specific instrumentation is not superior compared to conventional guides for, achieving a correctly positioned TKA in patients with mild deformity, in which none of the other indications for PSI or present. However, further studies using new PSI technologies and larger numbers of cases, evaluating radiological and functional outcomes with long follow-up, are important to assess the utility of PSI.

## Data Availability

All data is available, upon reasonable request, from the corresponding author.
